# Genetically modified foods: bibliometric analysis on consumer perception and preference

**DOI:** 10.1080/21645698.2022.2038525

**Published:** 2022-04-11

**Authors:** Sendhil R, Joan Nyika, Sheel Yadav, Joby Mackolil, Rama Prashat G, Endashaw Workie, Raja Ragupathy, P. Ramasundaram

**Affiliations:** a ICAR-Indian Institute of Wheat and Barley Research, Karnal, India; b Technical University of Kenya, Nairobi, Kenya; c ICAR-National Bureau of Plant Genetic Resources, New Delhi, India; d CHRIST (Deemed to be University), Bangalore, India; e ICAR-Indian Agricultural Research Institute, New Delhi, India; f School of Environmental science and Engineering, Shanghai Jiao Tong University, Shanghai, China; g Lethbridge Research and Development Centre, Agriculture & Agri Food Canada, Alberta, Canada; h National Agricultural Higher Education Project, Indian Council of Agricultural Research, New Delhi, India

**Keywords:** Bibliometric analysis, consumer perception, consumer preferences, GMOs, GM food, industry implications, policy imperatives, future research thrust

## Abstract

In this study, we present the bibliometric trends emerging from research outputs on consumer perception and preference for genetically modified (GM) foods and policy prescriptions for enabling the consumption using VOSviewer visualization software. Consumers’ positive response is largely influenced by the decision of the governments to ban or approve the GM crops cultivation. Similarly, the public support increases when the potential benefits of the technology are well articulated, consumption increases with a price discount, people’s trust on the government and belief in science increases with a positive influence by the media. Europe and the USA are the first region and country, respectively, in terms of the number of active institutions per research output, per-capita GDP publication and citations. We suggest research-, agri-food industries-, and society-oriented policies to be implemented by the stakeholders to ensure the safety of GM foods, encourage consumer-based studies, and increase public awareness toward these food products.

## 1. Introduction

Feeding the burgeoning population, estimated to reach 11 billion by 2100 AD, will undoubtedly be a herculean task.^1,2^ It is estimated that a vast majority of this population growth will occur in developing countries, home to more than two-thirds of those suffering from hunger. The Food and Agriculture Organization (FAO) estimated that 653 million people were undernourished in 2015 and the number rose to 690 million in 2019. It is expected that the number will continue to rise hence the difficulty in realizing an end to hunger and malnutrition by 2030.^2^ Timely policy intervention is required to liberate these economies from the shackles of hunger and poverty. With the finite arable land being exposed to high rates of soil and water degradation, cultivation of our future crops will be increasingly challenging. Sustainable food production under such circumstances, demands agricultural scientists across the globe to develop improved cultivars with enhanced productivity using modern tools of plant- breeding, production and protection. It is through the scientific efforts and farmers’ endeavors, that the annual average increase in yields to the tune of 1.2% has been achieved for the four staples, i.e., wheat, rice, maize and soybean, which together contribute to 66% of calorie intake in the global diet.^3^ This gain in yield still is lower than the normative growth rate of 2.4% per annum required to support the predicted global population.^4^ What lies ahead is a tumultuous path with challenges like climate change with frequent extreme weather events, emergence of new insect-pests and diseases, weeds, and yield plateau. This necessitates a multi-pronged strategy aiming at development of cultivars with increased productivity which are climate resilient, nutritionally superior, resistant to biotic stresses and leave a reduced ‘carbon’ footprint on the environment.

Crop improvement based on scientific principles dates back to the 18^th^ century.^5,6^ The significant role of breeders during the 1960s, in improving the productivity was very crucial one. The era of ‘green revolution’ heralded the emergence of hybridization and selective breeding, as drivers for enhancing food production through the development of high yielding semi-dwarf varieties, tailored for new mechanized cultivation practices and responsive to synthetic fertilizers that replaced low yielding multi crops with high yielding mono crops. However, conventional breeding has a major limitation in terms of utilizing genes from tertiary gene pools due to barriers arising from reproductive isolation. This is where GM technology, along with marker- and genomics- assisted crop improvement, strengthened accomplishment of sustainable global food security. GMO technologies offer a much wider scope, allowing gene introgression, overcoming the reproductive barriers defining the species. The World Health Organization (WHO) defines “genetically modified organisms (GMOs) as organisms (i.e. plants, animals or microorganisms) in which the genetic material (DNA) has been altered in a way that does not occur naturally by mating and/or natural recombination.” The foods which are derived from GM organisms are often referred to as GM foods. Since the development of the first transgenic plant for agriculture in 1983, the GMO technology has grown by leaps and bounds. These are cultivated across 26 countries on a global area of 191.7 million hectares.^7^ The three most common traits found in GM crops include herbicide tolerance, resistance to insects and plant viruses. The top five biotech crops grown are soybean, maize, cotton, canola and alfalfa driven by commercial farming and commodity values. For maize, as high as 137 transgenic events have been approved in over 35 countries.^8^ Countries like the USA, Brazil, Argentina and Canada are the major producers and exporters of GM crops and products (https://www.isaaa.org/). Owing to the phenomenal increase in area under GM crops, this technology has been claimed to have become the fastest adopted crop technology in the world, with a ~ 113-fold increase from 1996. However, the expansion in production and commercialization of GM crops has not been smooth, and was impeded with protests and bans by many governments worldwide, due to recommendations by various national regulatory bodies. While the impact of GM foods in enhancing crop productivity cannot be overlooked, a large section of the society remains wary of the possible adverse impacts of GM products.^9,^^10–17^ The reasons for this apprehension are many, some of which are the potentially adverse impact of GM food crops on human health and environment, lack of public awareness, lack of independent and credible scientific studies on risk assessment, and policy bottlenecks. Lack of consensus among the scientific community regarding the environmental and food safety issues is another major concern.^4,18^ There are several issues that have been intensely debated in many public and academic forums. The most prominent of these issues is the likelihood of gene flow to the wild and weedy relatives rendering them more aggressive, allergenicity in humans due to the new protein synthesized in the GM food crop, monopolization in food supply and vertical restraint, which defeats the very idea of consumer welfare.^19^ The safety of GM food for human consumption remains a topic of debate amongst scientists, policy makers and consumers. To allay the fears arising due to the presence of antibiotic selectable marker genes which have been feared to lead to the evolution of “superbugs” through horizontal gene transfer, a great deal of research has been undertaken toward the development of transformation methods which are marker-free and strategies for selectable marker elimination through homologous recombination, and transposition have been developed.

Due to differences in consumer responsiveness toward GM food crops, the regulatory framework adopted by different countries for their release and commercialization are significantly different. While countries like the USA and Canada, adopt a more flexible and receptive outlook toward the GM food crops, countries in Europe are more opposed when it comes to the environmental release of GM food crops. The approach of the USA for regulation of GMOs is based on the supposition that regulation should focus on the nature of the products (product-based), rather than the process involved in the production.^20^ In order to address the concerns raised by consumers, different labeling requirements are in place, in different countries. While the United States Food and Drug Administration (FDA), does not require labeling of GM foods unless the transgenic food is substantially different from its conventional counterpart, the EU, by contrast, mandates the labeling of all foodstuffs, additives and flavors, containing 1% or more genetically modified material [Regulations 1139/98 and 49/2000]^21^ India is still waiting for its first GM food crop (*Bt* brinjal) to be commercialized. It has been put on an indefinite moratorium since 2010. This casts shadows of uncertainty on the fates of many GM crops, food and non-food crops which are at different stages of development in the laboratories and field trials for release. Owing to the different policies adopted for regulation of GM food across the world, there are large perceivable differences in the research and developmental activities. This is demonstrated in terms of differences in the number of publications emerging from different countries with regard to GM food, the active involvement of funding agencies in facilitating the research and developmental activities. There are several such parameters which can be analyzed to understand the global perception of these crops and these can potentially serve as indicators to predict their future. Even though a large number of review articles is available where the authors have very diligently described the GMO technology and its pros and cons, the scientific, economic, environmental and cultural impact, an analysis of the vast amount of this information, in terms of bibliometric studies is rarely found. Similar analysis has been recently used to assess the research patterns concerning various health related topics such as incidence of diseases like malaria, tuberculosis;^22^ and medical data analysis.^23^ Considering the plethora of literature available on this topic, a bibliometric visualization analysis will allow assessment of the current research activities with regards to GM food development, estimation of relative contribution of different countries and will shed light on future developmental trajectories in this area. Using the information from the bibliometric review, we discussed the reasons behind varying adoption and diffusion rates of GM crops, divided consumer preference toward GM food intake, followed by socio-economic and political situations in deciding the policy formulation and implementation. The method has become very significant in evaluating research and productivity among professionals at community level and especially those in the non-library information science sector.^24^

## 2 Conceptual Design and Methodology

**Figure 1.**
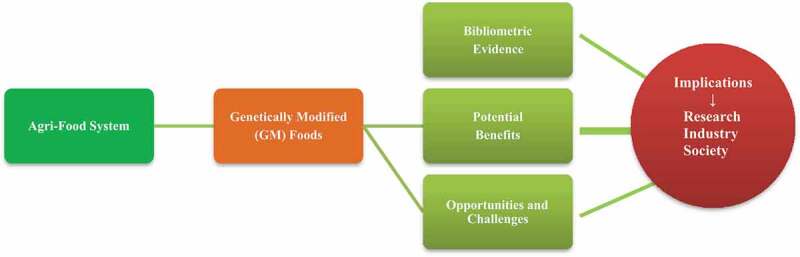
Conceptual design of the study.

GMO, despite being one of the fastest adopted technologies due to its scientific, economic and environmental merits, has faced opposition owing to the diverse regulatory mechanism arising from political ideologies and cultural perceptions leading to road blocks in universal consumer acceptance. The present study has been conceptualized to capture the bibliometric evidence of perception and consumer preference toward GM food using a visualization tool, its potential benefits to various stakeholders, opportunities and challenges followed by policy prescriptions for all researchers, stakeholders including agri-industries, producers and consumers (Fig. 1). The aim is to understand the progress and challenges from preexistent studies and from them device policy implications and prospects of the future regarding consumer preference to GM foods.

### 2.1 Database Selection

The Web of Science (WOS) database was used to retrieve relevant articles and data. Several advantages of WOS make it suitable for bibliometric analyses. It is one of the most widely used databases for searching publications and has a more consistent coverage of publications dating back to 1900. Besides, searches done on the database are robust, not limited to English language and in addition, include details of authors, citations, open access options and funding agencies.^25^ The database also gives access to archived information from journals according to Mongeon and Paul-Hus.^26^

### 22 Search Strategy

One of the pre-conditions of scientometric analysis is coming up with a good search query of high validity for comprehensive results and analysis. This study developed queries on the topic of GM food by doing a pre-review of both gray literature and scientific publications to compile some search phrases as advised by Sweileh and Mansour,^27^ who conducted a bibliometric analysis on environmental antimicrobial resistance. Four study scenarios joined by the Boolean operator “or” were used. The specific searches were “perceptions on genetically modified food” or “attitudes towards genetically modified food” or “preference rates of genetically modified food” or “consumer behaviour towards genetically modified food.” The options were searched on topic basis.

### 2.3 Refining Results Retrieved from the Search

Total documents retrieved were 616 consisting of 543 research articles (88.15%), 36 reviews (5.84%), 16 proceeding papers (2.60%), 10 early access (1.62%), 7 editorial material (1.14%), 3 book chapters (0.49%), 1 meeting abstract and news item each (0.16%). The cross- checking of the searched results through screening and eligibility analysis for missing data or false negatives led to exclusion of 28 and inclusion of 588 documents. The further search restricted to exclusively journal articles to focus more on original research, resulted to 543 journal articles, used for the final bibliometric analysis. The study period was 1981 to 2021, though initial evaluations showed limited research before the year 2000. Language restrictions were not imposed. The flow of bibliometric search is given in Appendix.

### 2.4 Data Export and Analysis

Retrieved data were transferred from WOS to Microsoft Excel for tabular and graphical representation. The VOS viewer software was used to create the visualization maps.^28^ Using the link strengths from the maps, different inter-relationships of the articles, countries and institutions of their origin, co-citations and authors were represented. A thicker connecting line and a higher value for the link corresponded to a stronger correlation of the aspect of evaluation.^28^ Several bibliometric indicators used in this study include: 1) growth in publication in the evaluation period, 2) research themes relevant to the search query, 3) most active journals, funding agencies, institutions and countries and 4) co-citations.

## 3 Bibliometric Evidence

### 3.1 Features of Publications Retrieved

**Figure 2.**
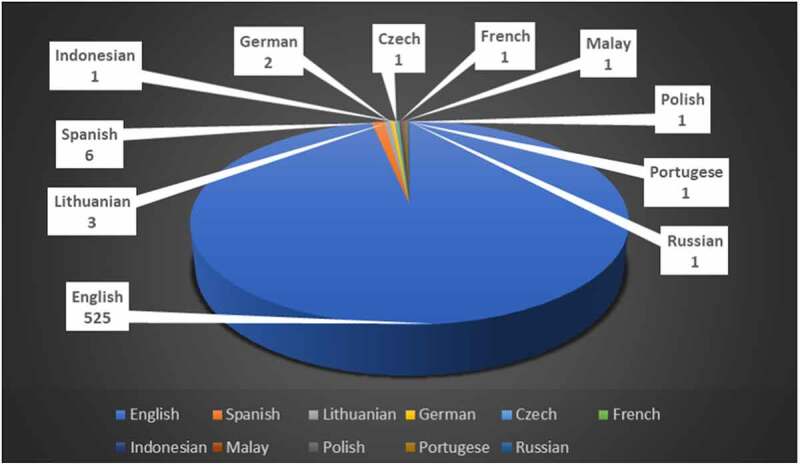
Distribution of retrieved articles by language.

n inputting the search query in the WOS database, 543 journal articles were retrieved that constituted the majority of the document type at 88.15%. While the majority of the articles was written in English (n = 525; 96.7%), as depicted in Fig. 2, the rest were written in Spanish (n = 6; 1.1%), Lithuanian (n = 3; 0.6%), German (n = 2; 0.4%), Czech (n = 1; 0.2%), French (n = 1; 0.2%), Indonesian (n = 1; 0.2%), Malay (n = 1; 0.2%), Polish (n = 1; 0.2%), Portuguese (n = 1; 0.2%) and Russian (n = 1; 0.2%). About 29% (n = 156) of the total retrieved journal articles were available as open access to the readers.

### 3.2 Annual Growth in Publications

**Figure 3.**
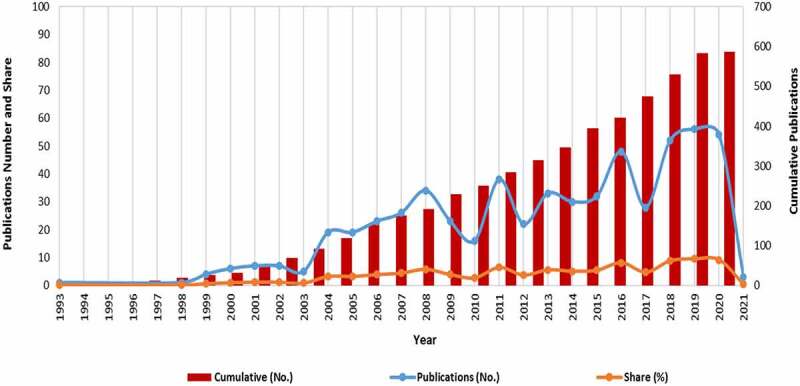
Annual growth in publications on perceptions, attitudes, preferences and consumer behavior on GM food (1981-present day).

The incremental growth in the searched area of GM food perceptions, attitudes, preferences and consumer behavior was on a rising trend based over the past five years (Fig. 3), compared to the period 1990–2005 when the number of publications recorded on the topic was limited.

### 3.3 Subjects and Category Analysis

Two categories were used to analyze the identified articles to understand the main subjects and research themes associated with the query. The first category was using the research areas generated by the WOS database, indicating common and broad areas including engineering, agriculture and other sciences associated with the search query. The second approach used the WOS categories that described sub-fields populated from classifications of the database. Overall, 58 research areas were identified based on the first approach. Of them, the top 20 along with their publications record were as shown in Table 1. Total related to WOS categories on the basis of the second approach were 86 and the top 20 were shown in Table 2. The top three research themes viz., Agriculture, Food Science Technology and Business Economics accounted for 62.8% (Table 1) and their respective identified categories viz., Food Science Technology, Agricultural Economics Policy and Economics constituting 51.2% were closely related (Table 2).

**Table 1.** Research areas identified in the WOS and their occurrence

No.	Research Areas	Record Count	Percentage
1.	Agriculture	126	23.2
2.	Food Science Technology	123	22.7
3.	Business Economics	92	16.9
4.	Environmental Sciences Ecology	56	10.3
5.	Nutrition Dietetics	56	10.3
6.	Communication	48	8.8
7.	Social Sciences other Topics	47	8.7
8.	Public Environmental Occupational Health	42	7.7
9.	Science Technology other Topics	41	7.6
10.	History Philosophy of Science	39	7.2
11.	Biotechnology Applied Microbiology	37	6.8
12.	Behavioral Sciences	25	4.6
13.	Mathematics	23	4.2
14.	Mathematical Methods in Social Sciences	22	4.1
15.	Psychology	16	2.9
16.	Engineering	15	2.8
17.	Biomedical Social Sciences	13	2.4
18.	Education Educational Research	11	2.0
19.	Biochemistry Molecular Biology	10	1.8
20.	Philosophy	9	1.7

**Table 2.** Identified WOS categories and their occurrence

No.	Web of Science Categories	Record Count	Percentage
1.	Food Science Technology	123	22.7
2.	Agricultural Economics Policy	88	16.2
3.	Economics	67	12.3
4.	Nutrition Dietetics	56	10.3
5.	Communication	48	8.8
6.	Public Environmental Occupational Health	42	7.7
7.	History Philosophy of Science	39	7.2
8.	Environmental Sciences	38	7.0
9.	Biotechnology Applied Microbiology	37	6.8
10.	Environmental Studies	29	5.3
11.	Social Sciences Interdisciplinary	28	5.2
12.	Agriculture Multidisciplinary	26	4.8
13.	Behavioral Sciences	25	4.6
14.	Multidisciplinary Sciences	24	4.4
15.	Business	23	4.4
16.	Mathematics Interdisciplinary Applications	22	4.1
17.	Social Sciences Mathematical Methods	22	4.1
18.	Ethics	17	3.1
19.	Green Sustainable Science Technology	15	2.8
20.	Social Sciences Biomedical	13	2.4

### 3.4 Keywords Analysis

**Figure 4.**
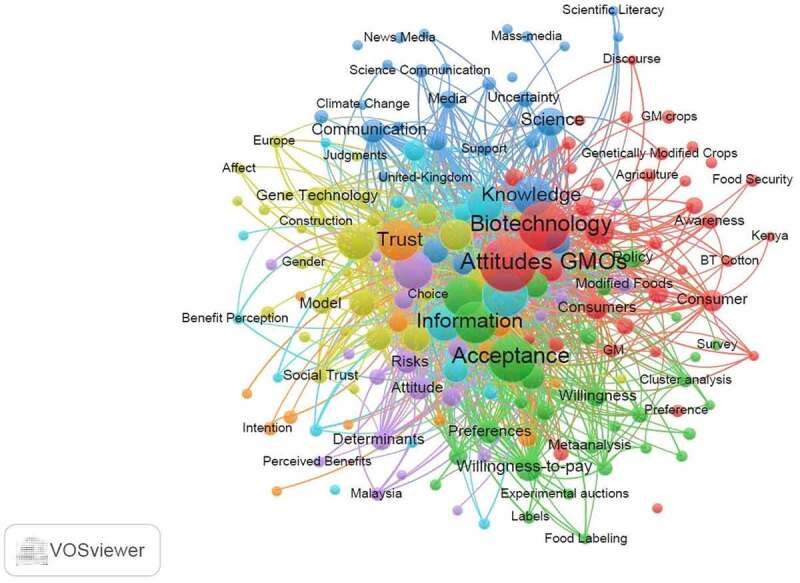
Bibliographic coupling of keywords.

The analysis of keywords is essential for detailing the themes of a given subject and identifying its research hotspots compared to other disciplines. According to Torres et al.,^29^ keyword analysis enables researchers to explore and focus on dominant research subjects and themes. Using the VOSviewer software, 2,150 words were identified from the downloaded documents and on using a threshold of five (*i.e*., at least five times the keywords get repeated across the searched articles), 178 keywords met the criterion. A bibliographic coupling of the resultant keywords is shown in Fig. 4.

Keywords with stronger links to each other included attitude, acceptance, biotechnology, perceptions, information, genetically modified foods, knowledge and benefits evident from the size of their circles. The observations corresponded to their frequency at 167, 118, 138, 110, 99, 90, 81, 86 and 69 times in respective order. The colors of the various keywords corresponded to their research themes and categories as classified by the WOS.

### 3.5 Geographical Distribution of Retrieved Documents

The most active publishers on the subject of GM food perceptions, attitudes, preferences and consumer behavior were the authors from European countries (n = 320) and United States (n = 161) followed by the African (n = 20) and Asian regions (n = 133) which contributed the least to the available literature. The top 15 publishing countries and their gross domestic product (GDP) per capita is presented in Table 3. The involvement in GMO research was favored in developed and fast developing countries with high per capita GDP.

**Table 3.** Top 15 countries involved in publications on GM food perceptions, attitudes, preferences and consumer behavior

Country	Frequency (%) N = 543	GDP per capita ($)
USA	138 (25.4)	62, 530
England	55 (10.1)	46,659
Peoples Republic of China	54 (9.9)	16,117
Australia	32 (5.9)	49,854
Netherlands	32 (5.9)	56,935
Canada	31 (5.7)	49,031
Germany	29 (5.3)	53,919
Italy	23 (4.2)	42,492
Spain	22 (4.1)	40,903
Malaysia	21 (3.9)	28,364
Denmark	19 (3.5)	57,804
Belgium	18 (3.3)	51,934
Switzerland	18 (3.3)	68,628
France	16 (2.9)	46,184
Turkey	15 (2.8)	28,424

### 3.6 Active Countries

**Figure 5.**
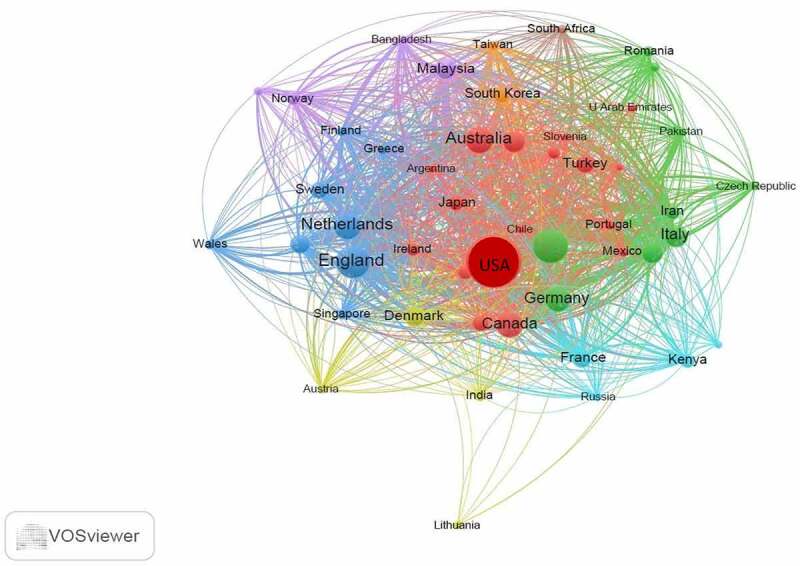
Bibliographic coupling of countries in research on GMO food perceptions, attitudes, preferences and consumer behavior.

The USA, England, China, Australia, Netherlands and Canada were some of the top publishers as shown in Table 3. The bibliographic coupling using the VOSviewer also interrelated these countries as shown in Fig. 5 based on occurrence of articles and citations. Sixty-seven countries were identified and by setting a threshold of 2 documents per country, 49 were included in the bibliographic coupling. Countries with the strongest linkages included USA, China, England, Netherlands and Spain based on their frequency of documents at 137, 54, 53, 32 and 22 times and citations of 3062, 562, 2197, 1335 and 524 times in respective order.

### 3.7 International Research Collaborations

**Figure 6.**
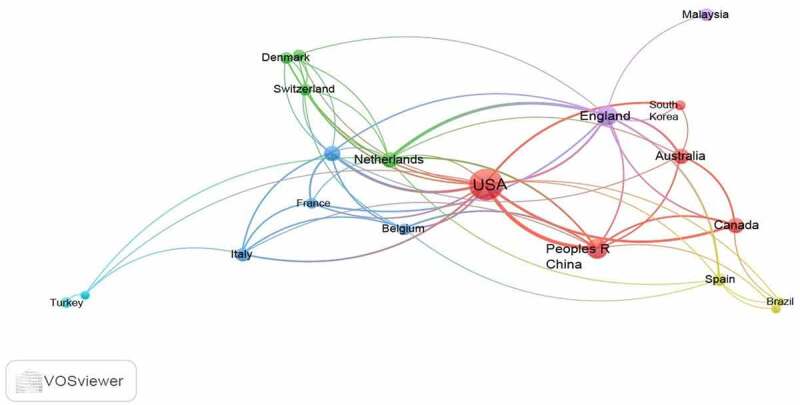
A network visualization map of research collaborations among countries with a minimum research output of 10 articles on GMO food perceptions, attitudes, preferences and consumer behavior.

Countries with a minimum of 10 articles were visualized on the VOSviewer to evaluate global research collaboration in the top publishing countries as shown in Fig. 6. Out of the possible 67 countries only 20 met this condition. Top five collaborators were USA, England, China, Germany and Netherlands with link strengths of 51, 33, 28, 21 and 21, respectively. Most countries recorded low link strength of <10, which was indicative of limited international research collaboration.

### 3.8 Citation Analysis

**Figure 7.**
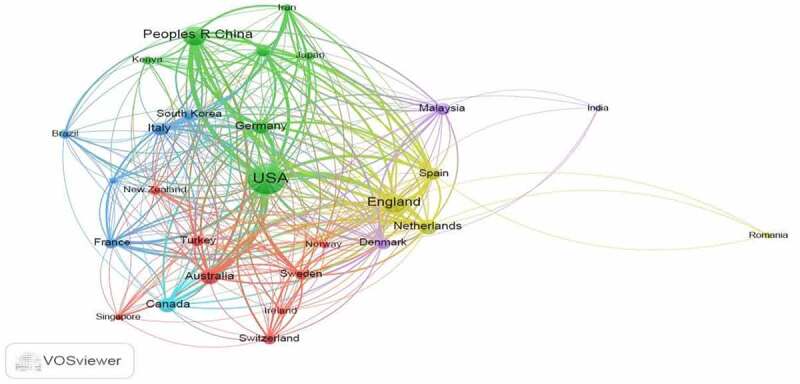
A network visualization map on the effect of publications from countries with a minimum of five articles.

The 543 identified articles received 11,306 citations with an average of 20.8 per article and a total h-index of 52. The sum of citing articles was 7,333 and 6,930 excluding self-citations. When a threshold of five documents for each country was used, 28 met the requirement and the USA, England, China, Spain and Netherlands with 3062, 2197, 562, 524 and 1335 recorded top citations and a link strength of 824, 692, 508, 383 and 354 respectively. The network visualization of the co-citations using the VOS viewer software was as given in Fig. 7.

### 3.9 Active Institutions

Six hundred and forty-five institutions were involved in publishing research of GMO food perceptions, attitudes, preferences and consumer behavior, and the top 10 publishing institutions are shown in Table 4 with Europe and USA having seven of them. The National University of Malaysia topped with 19 (3.5%) publications while Wageningen University Research and Ghent University followed with 16 and 14 publications, respectively (Table 4).

**Table 4.** Top publishing institutions on GMO consumption, perceptions and attitudes

Institution	Record Count	Percentage
The National University of Malaysia	19	3.5
Wageningen University Research	16	2.9
Ghent University	14	2.6
ETH Zurich	12	2.2
Chinese Academy of Sciences	11	2.0
Rutgers State University New Brunswick	11	2.0
University of North Carolina	11	2.0
AARHUS University	10	1.8
Michigan State University	10	1.8
CGIAR	9	1.7

**Figure 8.**
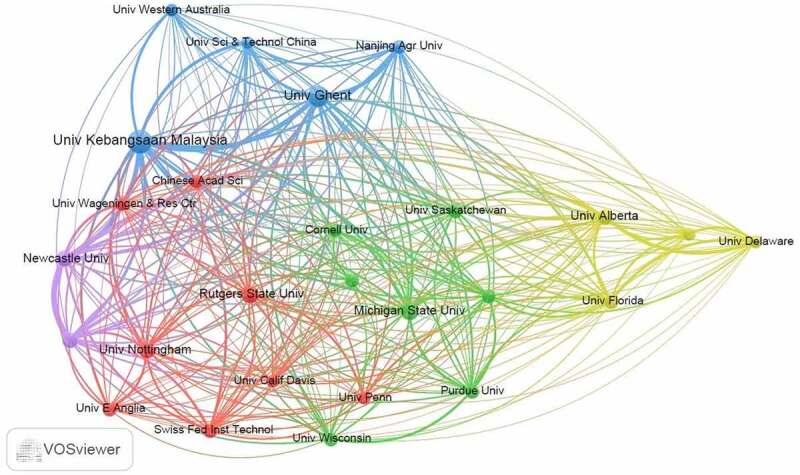
Bibliographic coupling of institutions involved in research on GMO food perceptions, attitudes, preferences and consumer behavior.

The bibliographic coupling of the various institutions using the VOSviewer is shown in Fig. 8. The minimum thresholds of 5 articles and 15 citations per organization were met by 26 institutions. The National University of Malaysia, Newcastle University, University of Ghent, Wageningen University Research and University of Alberta exhibited the highest interrelation with a total link strength of 2714, 2425, 2403, 1960 and 1294, respectively.

### 3.10 Active Journals

Top 10 active journals in publishing the research query related areas were as shown in Table 5. The most prolific journals were the ‘British Food Journal,’ ‘Food Quality and Preference’ and ‘Appetite’ with 5.5%, 4.6% and 4.25% respectively accounted for the highest total record count (n = 78). These top journals were drawn from the fields of food science technology and agricultural economics. Most of the journals had ranking by the Scopus at Q1 and their affiliations were mainly from the developed nations of Europe.

**Table 5.** Top 10 active journals in research on GMO consumption, perception and attitude

Source	Record Count	Percentage	Rank	Affiliation
British Food Journal	30	5.5	Q2	United Kingdom
Food Quality and Preference	25	4.6	Q1	United Kingdom
Appetite	23	4.2	Q1	Netherlands
Risk Analysis	22	4.1	Q1	United Kingdom
Journal of Risk Research	31	3.9	Q1	United Kingdom
Food Policy	18	3.3	Q1	United Kingdom
Public Understanding of Science	18	3.3	Q1	United Kingdom
African Journal of Biotechnology	11	2.0	0	Nigeria
PLOS One	11	2.0	Q1	USA
Sustainability	10	1.8	Q2	Switzerland

**Figure 9.**
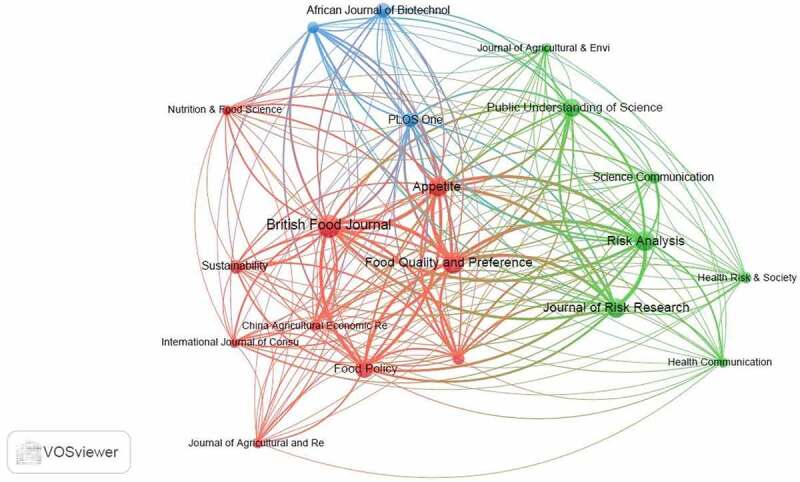
A network visualization on the bibliographic coupling of top sources with a minimum of five journal articles.

Out of the total 233 journals identified by the VOSviewer software, 20 met the threshold of a minimum of five articles for every source. Their bibliographic coupling is shown in Fig. 9. Journals that had a high number of documents had bigger circles and their link strength was higher comparatively.

### 3.11 Funding Agencies

Overall, 262 agencies were involved in funding the searched articles. The National Natural Science Foundation of China, The National University of Malaysia and National Science Foundation were the prominent funding agencies with a record count of 20 (3.7%), 16 (2.9%) and 9 (1.7%) affiliated documents, respectively. Other top funding agencies were as listed in Table 6.

**Table 6.** Top 10 funding agencies in research on GMO consumption, perceptions and attitudes

Funding Agencies	No. of Documents	Percentage
National Natural Science Foundation of China	20	3.7
The National University of Malaysia	16	2.9
National Science Foundation (NSF)	9	1.7
United States Department of Agriculture (USDA)	8	1.5
Economic Social Research Council (ESRC)	6	1.1
European Union (EU)	6	1.1
Genome Canada	6	1.1
National Institute of Health (NIH)-USA	6	1.1
United States Department of Health Human Services	6	1.1
CGIAR	5	0.9

### 3.12 Consumer Perception and Preference Toward GM Foods

Consumers experience anxiety toward GM food and therefore their decision on its consumption is pivotal to government and agri-business firms to prescribe policies and formulate strategies.^13,30^ A comprehensive information on consumers’ perception, preferences, attitude and response toward GM foods has been presented in the context based on the evidence from literature. A wide gap exists between acceptance for cultivation of GM crops and market across countries.^13^ Consumers’ knowledge plays a big role in influencing their attitude toward GM food purchase and consumption. For instance, a discernible attitude prevailed among the well-received consumers in the USA (without labeling) vis-à-vis EU (adopted the stringent approval and labeling).^31^ A majority of the respondents in the EU (61% in 2010) declined to support the GM foods and surprisingly 18% were not aware of the technology. The share of sample respondents not to support GM food was indeed lower (57% against the 61% in 2010) in the previous survey conducted five years before.^32^ In general, consumers in the EU have more negative perception and less purchase intention toward GM foods in contrast to the consumer perception in North America.^14,33–35^ Formal education and attitude toward GM foods seem to have low correlation among consumers in the Europe.^13^ Consumer behavior of purchase intention banked upon the risk and benefit perceptions^10,36^ is influenced by self-evaluation of product attributes attained through knowledge. For instance, purchase intention increases when the GM food sold at 15% discount with ‘spray-free GM’ label.^11^ Opinion is also divided on food safety between the American Association for the Advancement of Science researchers (88% supported) and general public (only 37% supported) with respect to consuming GM foods.^13^ Thus, trust is more important to convince general public. Compared to the USA and EU, in rest of the world with the exception of a few, data availability is a major concern.^33^ In developing nations, positive perception toward GM foods arises owing to the persistent demand for food and nutrition.^37^ Consumers in China, despite showing a positive attitude toward GM foods that have product-enhancing attributes, in the recent years have developed skepticism as more pondering discussions emerge on the consumption of GM foods.^12,38^ A survey of consumers (n = 2063) conducted in China revealed that 11.9, 41.4, and 46.7% respectively reported to have a positive, neutral, or negative perception on the GM food. Around 12% of the respondents claimed to be aware of the GM technology, while a majority were either ‘neutral’ or ‘unfamiliar.’ The major source of information was internet for a majority (69.3%) and 64.3% perceived that the information available on media is mostly negative toward GM food. Around 14% even perceived GM technology is a form of bio-terrorism on the country, despite a positive attitude expressed by the China’s Ministry of Agriculture and the science community.^39^ In a recent study in China on analyzing the perception and attitude of agri-business managers (n = 160) toward GM technology, it was found that they have a deep concern on GM food consumption and hence opposed the production.^40^ However, their attitude on GM crop farming is positively influenced by the level of expected profit and experience on GM crops research. Hwang & Nam,^10^ analyzed the influence of Koreans (n = 1000) knowledge on their perception and purchase intention toward GM foods and found that the imbalance between subjective and objective knowledge influenced their decision-making process. Low level of subjective knowledge undermined the consumers’ confidence on consuming GM foods. In addition, consequence-based consumers perspective leads to opposition.^41^ Apart from human consumption, even for livestock, from the literature evidence, the GM technology- based feed (*Bt* corn and/or roundup ready soybean) has not created any adverse effects on animal health.^42^

## 4 Potential Benefits of the Gm Crops

**Figure 10.**
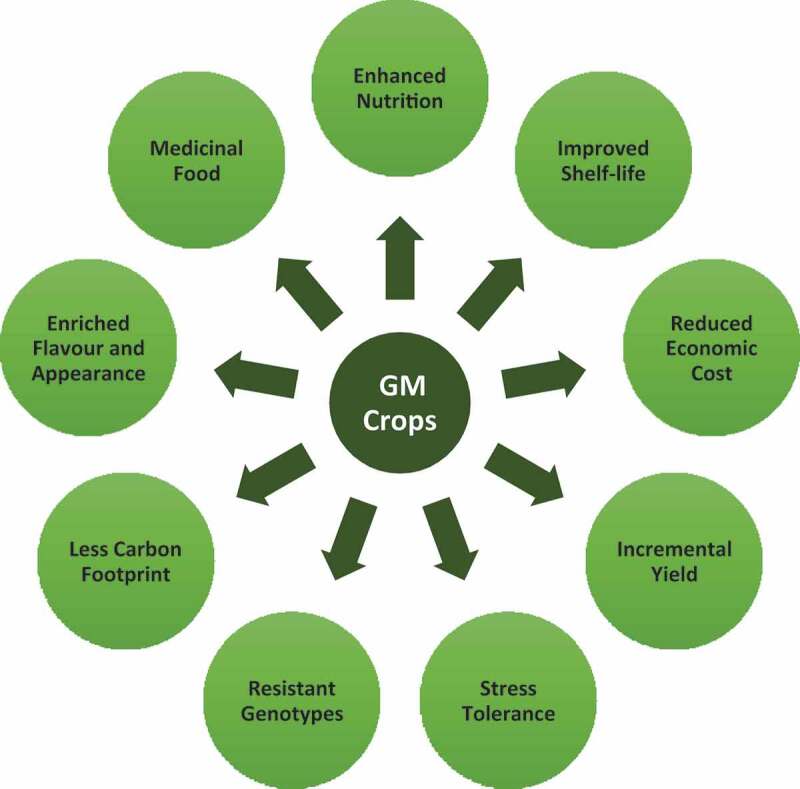
Potential benefits of the GMO technology in food crops.

Literature evidence is abundant on the potential benefits of GM technology in general and specifically in the food crops (Fig. 10). *Inter alia*, incremental yield gain is the prime benefit and target of the technology in food crops spurred by the spill over benefit in creating resistance to various biotic and abiotic stresses. For instance, soybean has been approved for commercial cultivation with the incorporated genetic change in herbicide tolerance to Glyphosate (Roundup). Similarly, *Bt* corn has been developed with resistance to insect pests, especially to the European corn borer.^43^ It has been estimated that the GMO technology has reduced the use of plant protection chemicals by 37%, resulting in increased yield to the tune of 22%^44^ and reduction in pesticides use by 8.2% in the past two decades,^1^^1.^https://www.bio.org/blogs/gmos-have-benefits-environment with an implicit benefit on cost reduction. Herbicide tolerant GM crops facilitate efficient weed control allowing for minimum soil tillage and erosion. In the aforementioned cases, the GM technology enables in protecting the environment owing to reduced chemical application. Also, evidence exist on GMOs benefiting the environment – reduction in CO2 emission equivalent to that of emission by 16.7 million cars in 2016 alone2. Similarly, the technology adds tolerance to abiotic stress like drought (in the case of wheat) and resistance to diseases, including late blight of potato, resulting in enhanced yield levels.^45–47^ A number of examples can be drawn on increase in the nutrition levels *viz*., beta-carotene rich rice [golden rice) as reviewed in Garg et al.,^48^ and a unique case like enriched flavor and appearance in the case of non-browning apple.^49^ Research also reports that the GM plants are expected to produce therapeutic recombinant protein and vaccines in the future.^43,50^ Further, in monetary terms, the GMO technologies earn super-normal profit to the seed producing companies that own the patent.

## 5. Challenges and Opportunities at the Forefront

Global food security is vital for the Sustainable Development Goals (SDGs) set by the United Nations.^51^ End to hunger, better nutrition, sustainable agriculture and achieving food security are of major focus in ‘SDG 2: Zero hunger.’ Improved agricultural techniques and food availability also have a key role to play even in SDG 1 that focuses on poverty reduction. Genetically modified food can significantly contribute to improved food security and mitigating undernutrition. Hence, the challenges associated with regulatory and consumer acceptance of GM food should be addressed with priority and care. This can be done by putting in place regulatory mechanisms and promoting campaigns on consumer awareness, sovereignty and rights over food safety. Possible measures must be adopted to transform the challenges into opportunities utilizing this proven tool to eradicate hunger and malnutrition. The cultivable land is scarce, especially in highly populated developing countries and the output per hectare for small-scale farmers is comparatively low. Irrespective of farm size, GM cotton for insect resistance was widely adopted.^52,53^ This encourages small-scale farmers to continue to grow GMO crops which can lead to enhanced farm income in developing countries. Hence, the challenge of dwindling cultivable land can be transformed into an opportunity by reverting to adopting more GM crops. The GM food crops require lesser amounts of chemical pesticides which makes it more sustainable and environment-friendly.^9^^,3,7,44,54^ Wu ^55^ reported a lower contamination of mycotoxins (*i.e*., toxic and carcinogenic chemicals produced as secondary metabolites of fungi), through pest protection, in the environment due to the adoption of GM technology. This also improves the quality of these products as the pesticide residue in the GM food products are relatively lesser. Golden Rice is a biofortified GM product with high beta-carotene. Similarly, GM foods have higher content of micronutrients like iron and zinc, highly beneficial especially in developing countries.^56–58^

GM food technology has a great possibility of developing products that possess the desired quality or nutritional value. For example, tomatoes with extended shelf-life and improved flavor and oilseed plants containing improved fatty acid profiles, attract consumers.^59^ But the substantial equivalence of the modified food product to conventionally developed products is important. The trait that is incorporated in the GM food product may lead to unintended side-effects which need to be assessed. For instance, GM foods with newly introduced proteins have reported potential allergenicity.^60^ Extensive research is yet to be carried out to produce a modified food product with superior properties and substantial equivalence but no significant side-effects. Emergence of efficient and accurate editing tools such as clustered regularly interspersed short palindromic repeats – associated proteins (CRISPR-Cas) has opened pathways to address the concerns of foreign gene introduction and off-target effects and commercialization of derived products.^61,62^

The labeling of products from GMO, particularly in Europe, have made these products stand out of the alternatives, raising the suspicion of the consumers and at the same time increased the cost involved. The separation of GM food products from non-GM food products was made mandatory in all stages of the production – from ‘field to fork.’^63^ In developing countries, this created an additional pressure as the labeling capacities are still in the nascent stage. Hence, many developing countries like Indonesia and India have to either remain GM-free or just continue with commercial GM crop production (like *Bt* cotton) instead of GM food crops^15^ https://www.isaaa.org/resources.

On the other hand, the consumer attitude toward genetically modified food products is still largely negative, particularly in EU and developing countries.^37,64,65^ Lack of consistent regulatory policies and approvals in spite of evidence about safety of GMO foods and sensational media reports strengthen consumer skepticism. Various issues like allergenicity, destruction of agricultural diversity, resistance to antibiotics, health risks are reported to be among the potential health and safety challenges.^59,66,67^ The gene flow through pollen leading to fertilization in other species is another concern raised. There is a lack of awareness among consumers and producers on GM food products. The commercialization and availability of GM foods is sub-optimal due to higher cost [$24.5 million for GM regulatory pipeline vs $10.5 million as conventional crop,^59^ lack of harmonized global regulation and mis- information by environmental interest groups.^64,68^ Collaborative initiatives by research labs with commercial firms is essential to develop GM food products. Awareness has to be spread among consumers regarding GM foods with science-based evidence to promote the products. Further, governments should formulate and implement necessary policies like compulsory labeling (short-term) by the producers, product-based regulation (long-term) and assure the public regarding the safety aspects besides facilitating sovereignty and protection of their rights over GM foods relevant for solving the hunger and nutrition issues.

## 6. Discussion

The current study is aimed at assessing the literature on consumer perceptions, attitudes, preferences and response toward GM foods. The preference and consumption to foods whose genetic composition has been modified by transformation remains controversial globally.^40^ Proponents of GM foods argue that it has positive impacts in addressing the global food insecurity amidst the climate change era and consequent economic growth.^17^ Conversely, the opponents believe it will alter the characteristics of native food (taste, appearance and texture), result in allergic reactions, cause harmful health effects and in the long-term, lead to environmental degradation. ^69^ From these viewpoints, the debate on the benefits, risks, perceptions, attitudes and consumer preferences on GMO foods are widely documented ^4,10,39^

An inventory on consumers’ decision on GM food becomes mandatory to formulate policies and strategies.^13,30^ Literature evidence alarms that the consumer response toward GM foods is largely negative, though the trend is changing in the recent past, especially post-implementation of stringent rules likes labeling. A majority of such studies have been carried out in the capitalist countries exhibiting a positive response, whereas, in the developing world, the literature is scanty and show a negative response toward the GM foods.^33^ Consumers positive response is largely influenced by the decision of the governments whether to ban or approve the GM crops cultivation.^70^ Similarly, the public support increases when the potential benefits of the GM technology are well articulated,^71^ consumption increases with a price discount,^11^ when people trust the government, believe science with a positive influence by the media.^37^ For instance, labeling as ‘spray-free GM’ fruits followed by 15% price discount in comparison to ‘organic’ fruits, the sales have increased from an experimental study reported by Knight et al.^11^ Singhal,^17^ listed that the consumption of GM foods is influenced by factors like the acceptance rate of the product, prevailing information, higher level of income and ethical consumption. In the recent years/studies, it is being articulated that the specialized researchers like biotechnologists with economics interest supports the technology by citing its potential benefits.^40^ The negative response, however, is largely attributed to the social stigma among consumers in buying the GM foods,^72^ media information,^39^ stringent regulations in production and trade,^15^ neophobia and psychological fear of health risk posed by innovative technologies and food products.^16^ Surprisingly, highly educated people with more income earning capacity along with higher level of food involvement as well as greater exposure to negative information tend to overestimate their actual knowledge level leading to a higher/lower level of risk/benefit perception, and lower intention toward the purchase of GM foods.^10^

Literature on GMO food perceptions, attitudes, preferences and consumer behavior is on a growing trend associated with recognition by international organizations and scientists as a viable, sustainable and safe solution to the global food insecurity indispensable in the era of climate change.^73^ The retrieved documents in this study were not only limited to food science technology, nutrition and dietetics but also span over other disciplines including agricultural economics, environmental sciences and applied genomics and microbiology to cite a few. A related scientometric analysis on GMO related research since 1995 to 2014 also concluded that the subject was multidisciplinary after identifying 117 subject categories affiliated to the search.^9^

The current study indicated that the USA, England and China are the leaders in publications on the searched query. China is one of the countries where food insecurity is on the rise due to a growing population with improved purchasing power by their largest segment of middle-class and demand for biofuels and feed.^74^ It is one of the leading countries to adopt GM technology for crop production and protection.^40^ Research by Wong and Chan,^75^ also noted that the USA, China and European countries were the hotspots for GMO application in agriculture. The inclusion of the USA, England and other European countries among the top publishers in the research is not a surprise given their research infrastructure with access to advanced technologies, abundant research funding and highly trained human resource *vis-à-vis* developing countries of Asia [with the exception of China) and Africa. Zhong et al. ^76^ reported a similar trend in a bibliometric analysis on natural resource accounting, which established that developed countries and fast-growing developing countries such as India and China had high numbers of published articles.

In the current study, two major research areas/ categories were evident in the analyzed literature: food science technology and agricultural economics. The former is a multidisciplinary field applying principles of analytical chemistry, quality control, food management and safety, engineering and biotechnology, all relevant to the searched query. Agricultural economics deals with optimizing crop and food production and distribution and concurrently enhancing safety to alleviate any consumer concerns in the processes. It is in the field of agricultural economics that the preferences, perceptions and consumer behavior toward GMO foods can be derived.

In this research, lead journals publishing impact of GM foods were from the food science technology, biotechnology and economics disciplines, mainly affiliated to the developed countries of Europe and the USA. This observation alludes to the fact that funding and investment on research and development is a determining factor of research output and improved scientific performance as highlighted by Ebadi and Schiffauerova.^77^ The suggestion also explains the limited research output from low-income developing countries whose investment on research and development is limited.^78,79^ In the present study, h-index and citations were indicative of wide readership in the searched subject. This trend could be associated with the optimism about GMO foods as their capacity to deliver toward future food security, and thus sustainable economies as projected by Gatew and Mengistu.^80^ Similarly, high readership on the searched GMO food aspects could be associated with the growing public awareness on the benefits and risks of consuming such foods as highlighted by Taheri et al.^81^ The high h-index associated even with low inter- country research collaboration attests to the relevance of the topic contrary to self-citations. The involvement of highly reputable journals in publishing on the topic could be associated with the high citations.

This is the first scientometric study using visualization tool on the consumer perceptions, attitudes and preferences toward GMO foods and the trends and growth on the topic since 1981 to 2021 using the WOS database. The use of the database to retrieve information on the topic excluding gray literature and non-indexed journal articles makes the conclusions drawn informative though preliminary, paving way for complex analysis. The term ‘GMO food’ is very general while other studies could be specific to a particular food and hence, not captured in the search. The work used the maximum known topics for the search but not all the possible ones. Despite these limitations, validation of the searched results minimized the possible errors of omission because articles could be retrieved, assessed and confirmed on their relevance with regard to the search query.

## 7. Conclusions and Policy Implications

In the attempt to capture the consumer perception and preference for GM foods through bibliometric analysis, we established the increasing trend in publication by retrieving 543 journal articles on the aforementioned topic. Thematic analysis indicated a strong interlinkage of GMO research with agriculture and food science technology. GMOs, biotechnology, attitudes and acceptance were identified as the most common keywords used in the topical research. Europe and the USA were the power houses in GM food research as captured by the factors like number of active institutions per research output, publication per GDP/capita and number of citations registered per article. British Food Journal, Food Quality and Preference, and Appetite have been identified as the preferred journals for the authors to publish their research output on GM food. Being a contemporary subject, a majority of the research publications were linked to the developed nations. The bibliometric analysis also indicated the escalating research outputs on GM food consumer’s acceptance and preferences despite a mixed opinion among the end-users, entailing the significance of future research thrust. The articles evaluated in the bibliometric analyses show that scientific communication on consumer perception and preferences to GM food is limited to active publishing regions while developing countries are not active.

### 7.1 Futuristic Research on GM Foods

The adoption of GM crops globally has impacted economically and environmentally *via* increased crop productivity and farmers’ income along with reduced cost and CO2 emission boosting stakeholders’ interests and environmental health.^7,44,54,69^

Despite the aforementioned issues, the GM crops are released for cultivation only with regulatory approval after stringent assessments for food and feed safety. The world area under transgenic crops has increased from 1.7 million hectares in 1996 to 191.7 million hectares in 2018, registering a 113-fold increase.^7^ The increase in area under GM crops has led to concerns regarding the food safety, environment and socio-economic issues. There are also concerns regarding the transgene flow into non-target species leading to feralization and its negative effects on biodiversity.^82–85^

The possibility of the selectable marker genes conferring antibiotic resistance transferring to human and animal gut microbes through GM foods and resulting in the development of antibiotic resistance was also raised as a concern.^86–88^ Efforts on having marker-free integration events to overcome this concern^89,90^ and genome editing of native *in-lieu* of introducing foreign DNA,^91^ were also successful.

The possible introgression of the transgenes from transgenic crops to wild relatives is a potential risk for loss of biodiversity and the gene flow to weedy relatives will lead to emergence of herbicide-resistant ‘superweeds.’ The pollen-mediated transfer of transgene from GM crops to traditional cultivars and to their wild relatives reported in maize, rice, cotton, barley, beans, creeping bent grass and rapeseed, is a major adverse effect on the environment.^92–96^

Extensive cultivation of insect resistant crops and the high selection pressure may lead to resistance in the targeted insect population causing emergence of new insect biotypes.^97,98^ The strategies involving the pyramiding of multiple insect-resistant genes and inclusion of susceptible host in cultivation as a refuge crop have been utilized successfully to delay the breakdown of resistance. The major limiting factors associated with the development and cultivation of transgenic crops are the high cost of safety assessment including containment facilities and the lengthy and complex regulatory approval process required before the commercial release.^99,100^ Regulatory approval post safety evaluations is the longest phase in the transgenic product development and commercialization^101^ and the estimated time is around five and seven years in European Union and the United States respectively.^102^

The scientific evidence on the environmental and health impacts of GMOs is still emerging and there is no conclusive evidence on the negative impacts. Though the perceptions of the public about GMOs in agriculture and food is divided in across developing and developed economies with an overall inclination toward avoiding GM food and products, the scenario is witnessing a discernible change. Governments all over the world are implementing various regulatory guidelines and policies to ensure safety of the consumers, producers, farm animals and the environment.

Regarding the international agreements, the ‘Cartagena Protocol’ on Biosafety came into force in September 2003, and by June 2020 has been ratified by 173 countries that aims to protect biological diversity from the potential risks posed by the GMOs. It establishes an advance informed agreement (AIA) procedure so that, countries are provided with the necessary information to make informed decisions before agreeing to the import of such organisms into their territory.^103, 104^

Public acceptance and proper policies are keys for agricultural, environmental and socio- economic benefits of transgenic crops to reach the poor and the needy. More important is the regional level regulatory harmonizations that facilitate data transportability for expediting the decision-making with regard to bio-safety. The benefits of the transgenic crops in the present scenario as well as in future depends upon science-based forward-looking regulatory steps, critically looking at the benefits rather than the risks, agricultural productivity with due considerations to environmental conservation and sustainability, and most importantly taking into consideration the millions of hungry and impoverished population.^7^

### 72 Policy Implications

In the light of the aforementioned review and discussion, we draw a raft of policy prescriptions for research, industries and society for a focused and pragmatic approach in GM food crops.

#### 7.2.1 Research

- Local governments enabling a favorable environment for R&D and outreach addressing the socio-political concerns and debates around GM foods/including gene edited crops.
- Invest and harness the potential of GM techniques including gene editing in crops for clean fuel production and biodegradation to combat the adverse climate change.
- Stringent, harmonized and universal protocols for testing the GM food based recombinant vaccines for public use.
- Harmonizing regulatory framework across the world *in lieu* of the current process- based regulation in EU countries and product-based regulation in North America, Argentina and Brazil.
- Bridging the gap between researchers’ and public opinion on GM food and safety through evidence-based studies.

#### 7.2.2 Agri-Food Industries

- Promoting public awareness on compliance of food safety standards and product labeling.
- Agri-food companies owning GMOs should disclose the critical technical details in the public domain allaying the apprehensions about their safety.
- GMO based food manufacturing companies have to warrant a safer and healthier food.
- Enabling the ‘traceability’ feature using the blockchain technology.

#### 7.2.3 Society

- Informed decision-making process in terms of scientific production as well as consumption.
- Belief and acceptance of evidence-based science than opinion-based misperceptions.
- Creating awareness on food safety protocols and food labeling.

Clearly, realization of GM technology in the agricultural food system needs due diligence and in-depth analysis on associated risks and/or benefits to multiple stakeholders on a case-to-case basis before commercialization.

## Supplementary Material

Supplemental Material
