# Supplementary material for: Genetically modified foods: bibliometric analysis on consumer perception and preference
Source: GM Crops Food. 2022 Apr 11;13(1):65–85. doi: 10.1080/21645698.2022.2038525 (PMC9009926; doi:10.1080/21645698.2022.2038525)
Supplement: Supplemental Material [file KGMC_A_2038525_SM4355.pdf]

543 Documents on GM Food

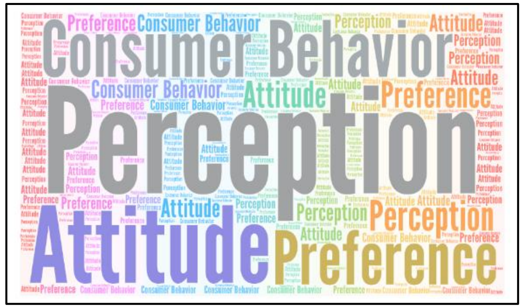

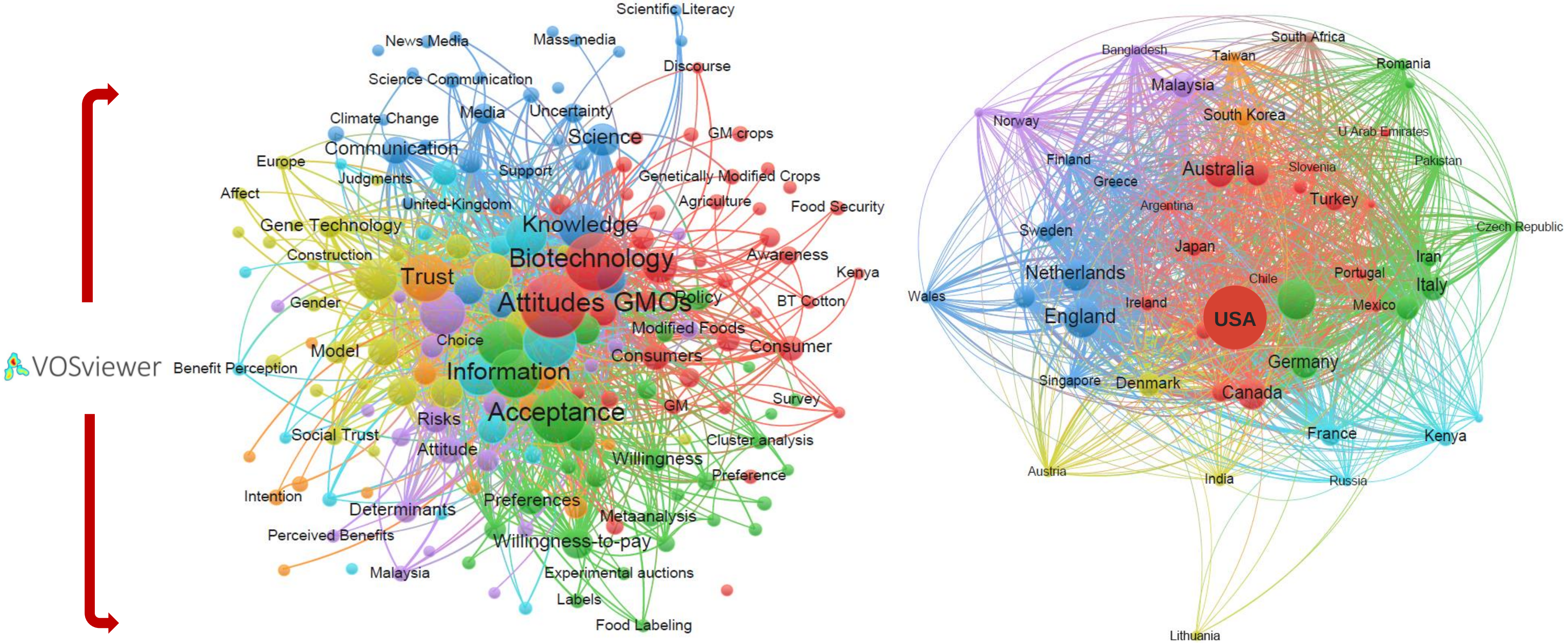

Bibliometric Evidence

- ❖ Agriculture and food science technology have a strong bibliometric linkage
- ❖ GMOs, biotechnology, attitudes & acceptance are the major recurrent keywords
- ❖ Europe and the USA lead in GMO research and collaboration
- ❖ Consumer acceptance and preference for GM foods are increasing
- ❖ Knowledge influences consumers' attitude towards purchase and consumption

Policy Imperatives

- ❖ Invest and align public R&D programs for leveraging the potentials of GM techniques
- ❖ Harmonizing the global regulatory framework
- ❖ Convergence of scientific and public opinion on GM food and safety
- ❖ Promote awareness of food safety standards and product labelling
- ❖ Enable informed decision in production and consumption
